# Modulation of Human Time Processing by Subthalamic Deep Brain Stimulation

**DOI:** 10.1371/journal.pone.0024589

**Published:** 2011-09-12

**Authors:** Lars Wojtecki, Saskia Elben, Lars Timmermann, Christiane Reck, Mohammad Maarouf, Silke Jörgens, Markus Ploner, Martin Südmeyer, Stefan Jun Groiss, Volker Sturm, Michael Niedeggen, Alfons Schnitzler

**Affiliations:** 1 Institute of Clinical Neuroscience and Medical Psychology, Heinrich-Heine University, Düsseldorf, Germany; 2 Department of Neurology, Medical Faculty, Heinrich-Heine University, Düsseldorf, Germany; 3 Department of Neurology, University of Cologne, Cologne, Germany; 4 Department of Stereotactic and Functional Neurosurgery, University of Cologne, Cologne, Germany; 5 Department of Neurology, Technische Universität, München, Germany; 6 Department of Educational Science and Psychology, Freie Universität, Berlin, Germany; Duke University, United States of America

## Abstract

Timing in the range of seconds referred to as interval timing is crucial for cognitive operations and conscious time processing**.** According to recent models of interval timing basal ganglia (BG) oscillatory loops are involved in time interval recognition. Parkinsońs disease (PD) is a typical disease of the basal ganglia that shows distortions in interval timing. Deep brain stimulation (DBS) of the subthalamic nucleus (STN) is a powerful treatment of PD which modulates motor and cognitive functions depending on stimulation frequency by affecting subcortical-cortical oscillatory loops. Thus, for the understanding of BG-involvement in interval timing it is of interest whether STN-DBS can modulate timing in a frequency dependent manner by interference with oscillatory time recognition processes. We examined production and reproduction of 5 and 15 second intervals and millisecond timing in a double blind, randomised, within-subject repeated-measures design of 12 PD-patients applying no, 10-Hz- and ≥130-Hz-STN-DBS compared to healthy controls. We found under(re-)production of the 15-second interval and a significant enhancement of this under(re-)production by 10-Hz-stimulation compared to no stimulation, ≥130-Hz-STN-DBS and controls. Milliseconds timing was not affected. We provide first evidence for a frequency-specific modulatory effect of STN-DBS on interval timing. Our results corroborate the involvement of BG in general and of the STN in particular in the cognitive representation of time intervals in the range of multiple seconds.

## Introduction

Time is a fundamental dimension of human existence. Up to date time research is one of the fields in cognitive neuroscience with many unsolved issues and competing theories about how the human brain processes time.

In a classical concept three crucial time scales have been proposed for different aspects of life. First, circadian timing in the range of 24 hours controls the sleep-wake rhythm [1] which depends on hypothalamic structures [2]. Second, milliseconds (ms) timing is crucially involved in motor control especially of precise discontinuous repetitive automatic movements [3] and relies on the cerebellum [4]. Third, timing in the range of (multiple) seconds (s) referred to as interval timing is essential for cognitive operations such as decision processes and conscious time processing and depends on a neural system involving frontoparietal cortices and basal ganglia (BG) [5], [6].

Parkinsońs disease (PD) is a neurodegenerative disease characterized by akinesia, rigidity and tremor resulting from a dopaminergic cell loss in the substantia nigra. In addition to motor deficits, PD patients show distortions in interval timing that can be relieved by L-dopa [7], [8], [9], [10]. Besides dopaminergic therapy deep brain stimulation (DBS) of the subthalamic nucleus (STN) is a powerful treatment of PD [11], [12]. DBS does not only improve motor functions but also influences cognitive and executive functions [13] by affecting non-motor loops of the STN [14], [15], [16], [17], [18]. With respect to interval timing DBS ameliorates the PD-associated impairment in memory retrieval of time intervals termed “memory migration effect” [19], [20]. This effect describes a phenomenon, where representations for different time lengths migrate towards each other in memory in such a manner that long intervals are estimated shorter whereas short intervals are estimated longer during retrieval.

Classical explanations of time perception using a pacemaker-accumulator model (scalar timing theory) [21] have been supplemented by a recent proposal, alleging that interval timing relies on the detection of coincident neuronal oscillations in subcortical and cortical circuits (striatal beat frequency model (SBF) [22]). According to this model thalamo-cortico-striatal loops are involved in time interval recognition such that striatal basal ganglia neurons detect specific oscillatory activation patterns of frontal cortical areas during time encoding into working memory. Interestingly, pathological alterations of neuronal oscillations have recently been implicated in the pathophysiology of PD symptoms [23], [24], [25], [26], [27]. Furthermore, recent studies suggest that STN-DBS differentially modulates motor and non-motor functions depending on the stimulation frequency [28], [29] probably by affecting subcortical-cortical oscillatory loops.

Taking the STN-pathways as a model for SBF in human time perception we therefore investigated the influence of STN-DBS at different stimulation frequencies on time interval perception and production at various timescales in four different paradigms. A double blind, randomised, within-subject repeated-measures design was used to investigate and compare the effects of STN-DBS at ≥130 Hz, 10 Hz, and no stimulation. For interval timing 5 and 15 s time production and memory dependent reproduction tasks were performed. For millisecond timing an unpaced tapping task and a time discrimination task with deviance intervals ranging from 80 to 400 ms were used. Millisecond timing tests were performed to comprehend differential stimulation effects on BG versus other (e.g. cerebellar) timing aspects. Reaction time tasks were performed to control for potential bias of motor performance on time judgements. Motor symptoms were assessed using the Unified Parkinsońs Disease Rating Scale (UPDRS) motor score [30]).

## Materials and Methods

### Participants

12 patients with advanced Parkinsońs disease (mean age 64 years, SD 8, range: 47–72; 6 male, 6 female) with implanted deep brain stimulation devices participated in the study. 12 age and sex matched healthy subjects (mean age 66, SD: 5, range 56–74 years; 6 male, 6 female) served as a control group. Participants gave written informed consent according to the Declaration of Helsinki. The local ethics committee (Ethics committee of the Medical Faculty, Heinrich-Heine University, Düsseldorf) gave its approval for the examination of deep brain stimulated patients with Parkinsońs disease using timing paradigms and using low-frequency DBS settings.

All participants had a Mattis Dementia Rating Scale (MDRS[31]) score ≥ 130 and a Beck Depression Inventory (BDI [32]) score ≤11, thereby excluding relevant cognitive decline or depression. Tables 1, 2 illustrate clinical features and scores of the PD-patients and controls.

**Table 1:** Patient characteristics with sex, age, disease duration, daily anti-parkinson medication, months since implantation in the subthalamic nucleus, disease type, predominant side, MDRS and BDI scores.

Patient/Sex/Age (years)	Disease Duration(years)	Medication (mg/day)	Months Since Implantation	Disease Type	Predominant Side	MDRS	BDI
1/M/46	13	8 Cabergoline, 550 L-Dopa, 600 Entacapone	42	T	L	144	4
2/F/65	12	1,5 Pramiprexole, 100 L-dopa, 400 Entacapone	19	HR	L	142	3
3/F/69	25	0,27 Pramipexole, 350 L-Dopa,1000 Entacapone, 150 Amantadine	20	T	R	143	9
4/F/61	11	6 Cabergoline, 775 L-Dopa, 300 Tolcapone	60	T	L	140	3
5/F/73	32	0,54 Pramipexole, 700 L-Dopa,100 Amantadine	96	HR	L	142	2
6/M/69	20	1,05 Pramipexole, 500 L-Dopa	41	T	L	138	3
7/M/66	17	6 Cabergoline, 750 L-Dopa, 1400 Entacapone, 250 Amantadine, 1 Rasagiline	73	HR	L	134	10
8/M/71	21	2,25 Ropinirole, 550 L-Dopa	70	HR	L	138	4
9/M/51	16	4 Cabergoline, 300 L-Dopa, 200 Entacapone,1 Rasagiline	63	HR	L	142	2
10/M/72	20	15 Ropinorole, 400 L-Dopa, 400 Entacapone,1 Rasagiline	17	HR	L	140	4
11/F/72	16	1,58 Pramipexole, 750 L-Dopa,1000 Entacapone, 1 Rasagiline	27	HR	L	141	3
12/F/61	20	2,1 Pramipexole, 350 L-Dopa, 1 Rasagiline	25	HR	L	143	5

**Table 2:** Control characteristics with sex, age, MDRS and BDI scores.

Control/Sex/ Age (years)	MDRS	BDI
1/F/56	143	0
2/M/63	144	1
3/F/66	142	5
4/M/62	144	3
5/F/69	144	3
6/F/64	136	4
7/M/74	141	6
8/F/63	138	2
9/M/65	141	7
10/F/71	140	0
111/M/70	142	3
12/M/65	135	2

Abbreviations: MDRS  =  Mattis Dementia Rating Scale; BDI  =  Beck Depression Inventory; HR  =  hypokinetic-rigid; T  =  tremor dominant; L  =  left; R  =  right.

### Deep Brain Stimulation

All patients had undergone surgery for bilateral implantation of stimulation electrodes (Model 3389, Medtronic, Minneapolis, MN, USA) in the STN at least one year prior to study enrolment to prevent bias due to the micro-lesion effect. During the study the active contacts, stimulation amplitude and pulse width parameters optimized for antiparkinson therapy were used (see Table 3). Stimulation parameters were kept constant except for frequency. Frequency of stimulation was changed between ≥130 Hz, 10 Hz and no stimulation (“OFF”) (see below).

**Table 3:** Stimulation parameters used for long term stimulation and active stimulation contact (monopolar, with impulse generator used as anode) for each hemisphere.

Patient	Amplitude (V)		Pulse Width (µs)		Frequency (Hz)		Contact	
	Left	Right	Left	Right	Left	Right	Left	Right
1	3,0	3,0	60	60	150	150	2	6
2	2,2	2,4	60	60	130	130	1	5
3	2,4	2,45	60	60	130	130	3	5
4	3,1	2,5	60	60	150	150	6 and 7	00
5	1,3	3,0	60	60	130	130	1	3
6	3,8	3,8	90	120	180	180	2	4
7	4,0	1,5	60	60	130	130	4	1
8	3,4	3,4	60	60	130	130	2	5
9	3,3	2,7	60	60	130	130	2	5
10	2,6	3,6	60	60	130	130	1	5
11	3,0	3,0	60	60	130	130	3	7
12	3,2	3,9	60	60	130	130	7	3

Abbreviations: V  =  Volt; µs  =  Microseconds; Hz  =  Hertz.

**Figure 1:**
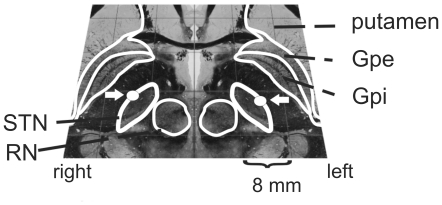
Stimulated area. Mean location of active contacts highlighted and marked with a white arrow at axial slice 3.5 mm under MCP of the Schaltenbrand and Wahren Atlas. Mean coordinates ± standard deviation were: right hemisphere: x-coordinate  = 13.7±1.7, y-coordinate  = −0.5±2.1, z-coordinate  = −2.4±2.0; left hemisphere: x-coordinate  = 13.0±1.3, y-coordinate  = −0.3±2.3, z-coordinate  = −2.8±2.8 Figure is based on the Cerefy Clinical Brain Atlas [53]. Abbreviations: STN  =  Nucleus subthalamicus; Gpe  =  Globus pallidus pars externus; Gpi  =  Globus pallidus pars internus; RN  =  Nucleus ruber; SN  =  substantia nigra.

To localize active contacts used for chronic stimulation postoperative stereotactic x-rays of 10 patients were available for reimport into the stereotactic planning system. Mean active contact position relative to the middle of the line between the anterior- and posterior-commissure (mid-commissural point, MCP) was calculated and visualized on the Schaltenbrand and Wahren Atlas [33]. As Figure 1 illustrates the mean active contact localisation was at the dorsolateral border of the STN.

### Design

A double blind randomised and within-subject repeated-measures design was used to investigate and compare the effects of DBS at ≥130 Hz, 10 Hz and no stimulation on time processing in PD-patients. Time processing at different time scales was assessed in four different paradigms. For interval timing a memory dependent time reproduction task and a time production task for intervals of 5 and 15 s length were performed. For millisecond timing a tapping task with inter-tap intervals of 800 ms and a time discrimination task with deviance intervals ranging from 80 to 400 ms were used. Reaction time tasks were performed to rule out bias on time judgements by motor deficits. Motor symptoms were assessed using the UPDRS motor score.

### Procedure

**Figure 2:**
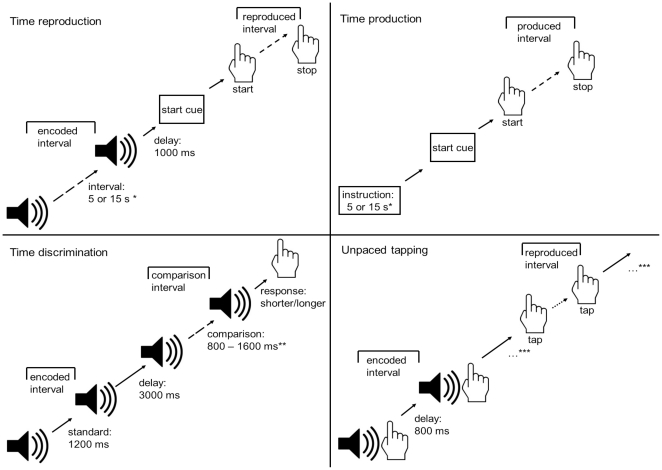
Paradigms. Illustration of the paradigms for time reproduction, time production, time discrimination and tapping. *10 cycles per interval, total of 20 trials; **10 steps of 80 ms, 5 cycles per interval, 10 cycles per each deviance (80, 160, 240, 320, 400 ms) from standard interval, total of 50 trials; ***total of 20 trials.

All tests were performed at the Department of Neurology of the University Hospital in Düsseldorf. Patients were tested without medication after 12 hours of dopaminergic medication withdrawal. The three deep brain stimulation conditions 10 Hz, “OFF” and ≥130 Hz were programmed directly without turning the device off between sessions in randomised order and kept constant for 15 minutes before starting the tests. In every stimulation condition all test were conducted within one block and in the same sequence. Motor examinations were performed by a blinded movement disorder specialist and videotaped. Time processing tests were initiated after careful oral and written instruction by a neuropsychologist and after a short training session. All tests were performed on a personal computer using E-Prime (Psychology Software Tools, Inc., Version 1.0 for Windows 98). For illustration of tests see Figure 2.

### Interval timing

#### Time reproduction

A tone (700 Hz, 2000 ms duration) was presented at the beginning and end of the 5 s or 15 s intervals and subjects were instructed to encode the intervals duration. These two test intervals were presented in random order. After a delay of 1 s the subjects were instructed to reproduce the interval by two button presses, one at the beginning and one at the end. After reproduction of the interval subjects were instructed to start the next trial by pressing a button. Each interval was presented 10 times. Relative deviations from the target interval were calculated.

#### Time production

An instruction on the computer screen requested the subjects to produce an interval of 5 s or 15 s. The subjects were not taught how long 5 s or 15 s intervals were. After a start cue the instruction was cleared from the screen and the time interval between two button presses was measured, marking the beginning and end of the produced interval. After a delay of 3 s the subjects could start the next trail by pressing a button. 5 s and 15 s intervals were each requested 10 times in a randomised order. Relative deviations from the target interval were calculated.

### Millisecond timing

#### Time discrimination

A tone (700 Hz, 200 ms length) was presented at the beginning and end of a standard interval of 1200 ms duration. After a delay of 3 s a comparison interval was presented in the same manner. The comparison interval had a length between 800 and 1600 ms, the length varying in steps of 80 ms (800, 880, 960, 1040, 1120, 1280, 1360, 1440, 1520, 1600 ms). Each comparison interval was randomly presented five times, resulting in 10 trials per deviance (80,160,240,320,400ms) from the standard interval, rendering a total of 50 trials. Subjects were instructed to judge if the comparison interval was longer or shorter than the standard interval by pressing respective buttons. The number of judgements “longer” and “shorter” were saved.

#### Tapping

The finger tapping task consisted of one run with two phases. First, subjects performed an auditory paced tapping task. Tones (700 Hz, 20 ms length) with an inter stimulus interval of 800 ms were presented and subjects were instructed to press a button with the onset of each tone. Second, after 20 auditory paced taps the tones stopped and the subjects were instructed to continue tapping at the given interval for 20 further taps without the pacer. The intertapping interval in the unpaced tapping phase was measured.

#### Reaction time

A tone (700 Hz, 1000 ms duration) was presented at a randomised interstimulus interval between 1 and 5 s. The participants were instructed to react to the tones as fast as possible by pressing a button. 20 trials were recorded.

### Statistical analysis

Measured time intervals and relative deviation from the target intervals for reaction time, reproduction, production and tapping, correct judgements for time discrimination and results of the motor scores were analysed with SPSS for Windows (SPPS Inc., Version 12.0). Considering the small sample size and as testing with the Kolmigorov-Smirnof test failed to show normal distribution for most samples nonparametric test were used to compare results between stimulation conditions within the PD group and between the PD patients and healthy controls. Friedman tests for related samples were used to analyse the effect of the factor “stimulation setting” within the PD-group. If a significant difference between stimulation conditions was detected, sequential Bonferroni corrected Wilcoxon-tests were performed for post hoc comparisons. To compare stimulation and control groups sequential Bonferroni corrected Mann-Whitney-U-tests for unrelated samples were used.

In the discrimination tasks a measure of the comparison duration judged equal to the standard interval, the point of subjective equality (PSE), and additionally as a measure of the precision of temporal discrimination, the just noticeable difference (JND), was determined. Thus, binomial logistic regression functions were fit to the data of each patient in all stimulation conditions and for control subjects. Fitting was performed with GraphPad Prism 5 (GraphPad Software, La Jolla California, USA). Two patients and two controls were excluded from analysis of PSE and three patients and controls were excluded from analysis of JND due to ambiguous fits. The duration with 50% “longer” judgments was taken as PSE. JND was calculated by taking the duration with 75% “longer” judgements minus the duration with 25% “longer” judgements divided by two. Statistical comparison of PSE and JND was then again done within the PD group using Friedman test and between controls and patients using Whitney-U-Tests.

## Results

### Interval timing: Time reproduction and production

**Figure 3:**
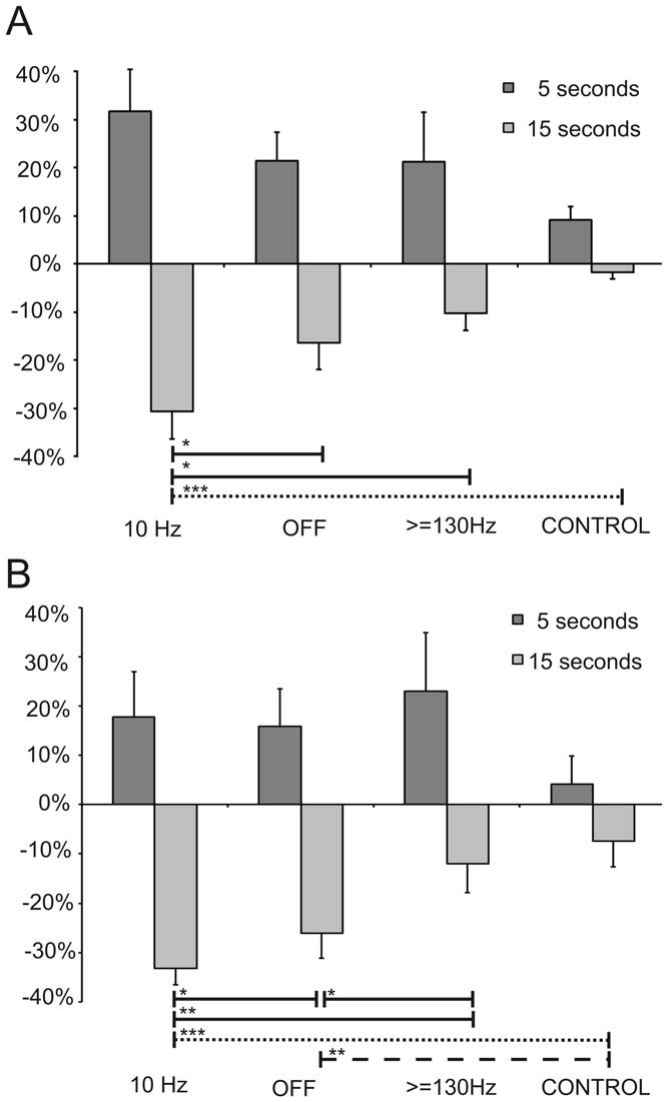
Mean results of interval timing. A: Mean results of time reproduction; B: Mean results of time production. Mean relative deviation (with SEM) from the target interval of 5 and 15 s for controls, PD-patients with stimulation OFF, > = 130 Hz and 10 Hz. Significant differences: *p< = 0.05; **p< = 0.01, ***p< = 0.001. Comparisons: continuous line: stimulation effect within PD group, dashed line: disease effect.

Patients and controls over-(re-)produced the 5 s interval and under-(re)produced the 15 s interval in both tasks (Figure 3 A and B).

For the 5 s interval in the production task there was no significant difference between controls and stimulation conditions or between the individual stimulation conditions after Bonferroni correction. In the 5 s reproduction task there was a trend revealing a difference between controls and patients with 10 Hz stimulation (p = 0.06) and without stimulation (“OFF”) (p = 0.07)).

Comparisons of the different stimulation conditions and healthy controls in the reproduction task for the 15 s interval showed that 10 Hz stimulation significantly enhanced the 15 s underreproduction effect (10 Hz: 10.4 s± (SEM) 0.9 s; compared to OFF: 12.5±0.8 s, p<0.05; compared to > = 130Hz: 13.5±0.6 s, p<0.05; compared to controls: 14.8±0.2 s, p<0.001). Correspondingly, in the 15 s production task underproduction was stronger with 10 Hz (10.1±0.5 s) than without stimulation (11.1±0.8 s, p<0.05), > = 130Hz stimulation (13.2±0.9 s; p<0.05) and than in controls (13.9±0.7 s, p<0.01). Furthermore the stimulation OFF differed from > = 130Hz (p<0.05) and normal controls (p<0.01).

Taken together, 10 Hz DBS significantly worsened interval timing at the 15 s interval and -discriptivly saying - controls and patients with > = 130Hz DBS showed lowest impairment of time processing. Furthermore, controls and patients with > = 130 Hz stimulation performed the 15 s time production significantly better compared to OFF stimulation. (Figure 3 A and B).

These differences in the production task between OFF and controls can also be interpreted as a *disease effect* (see dashed line in Figure 3 B). Correspondingly, the other differences can be named as a *stimulation effect* within the PD group, namely between 10 Hz vs. OFF and vs. 130 Hz in the reproduction task and in the production task between 10 Hz vs. OFF and vs. 130 Hz and additionally between 130 Hz vs. OFF (see continuous line in Figure 3A/B).

**Figure 4:**
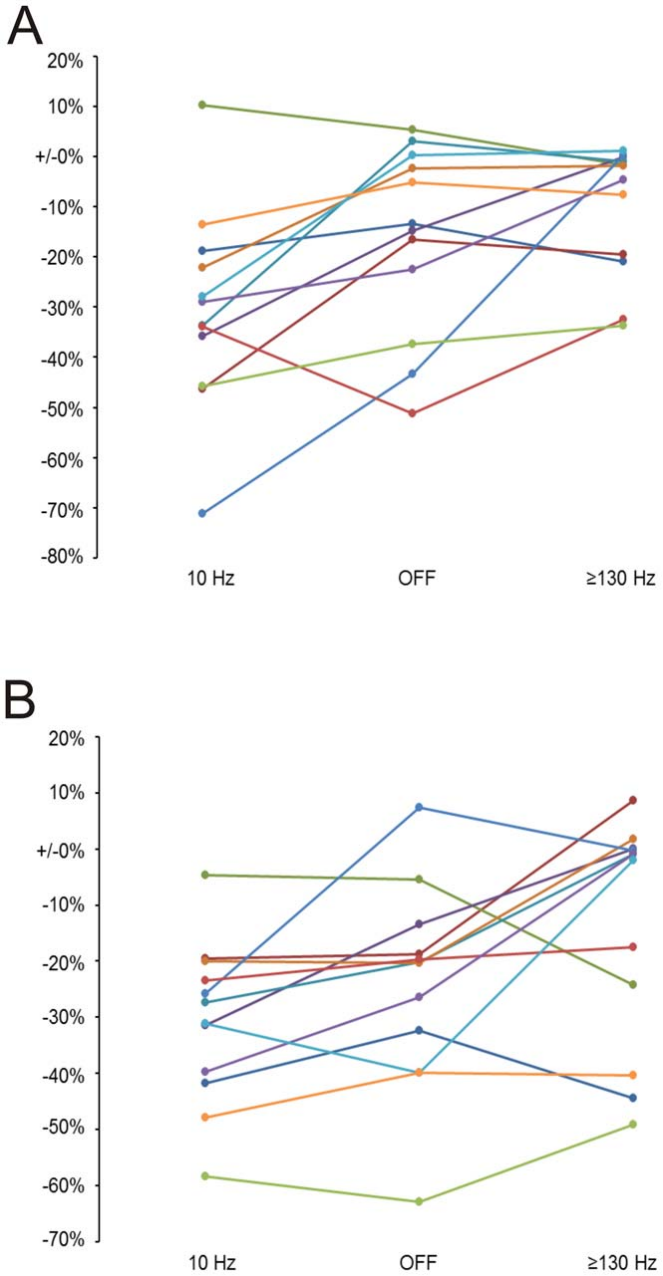
Individual results of interval timing. A: Indivdual time reproduction in DBS patients B: Individual time production in DBS patients. Individual relative deviation from the target interval for each PD patient and respective stimulation settings.

Furthermore, as Figure 4 illustrates, these *stimulation effects* within the patient group could be found in most of the subjects, with the most pronounced under(re-)production during the 10 Hz stimulation.

### Milliseconds timing: Time discrimination and tapping

**Figure 5:**
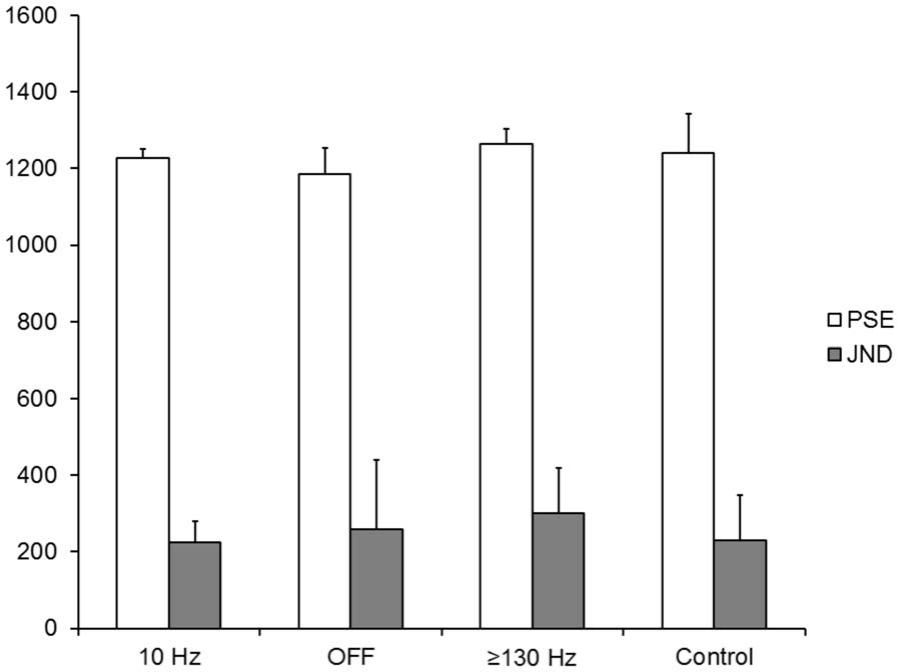
Mean results of time discrimination. Point of subjective equality (PSE) and just noticeable difference (JND) in ms (with SEM) for controls, PD-patients with stimulation OFF, > = 130 Hz and 10 Hz.

In the time discrimination task number of correct judgements for comparison intervals did not significantly differ between controls (41±2) and patients or between different stimulation conditions (10 Hz: 38±1; OFF: 35±2; > = 130 Hz: 35±2;). Furthermore PSE and JND did not differ significantly (PSE in ms:10 Hz: 1229 ±22; OFF: 1185 ±67; > = 130 Hz: 1265 ±40; controls: 1241± 102; JND in ms: 10 Hz: 226 ±55; OFF: 259 ±181; > = 130 Hz: 300 ±120; controls: 231± 118) (see Figure 5).

**Figure 6:**
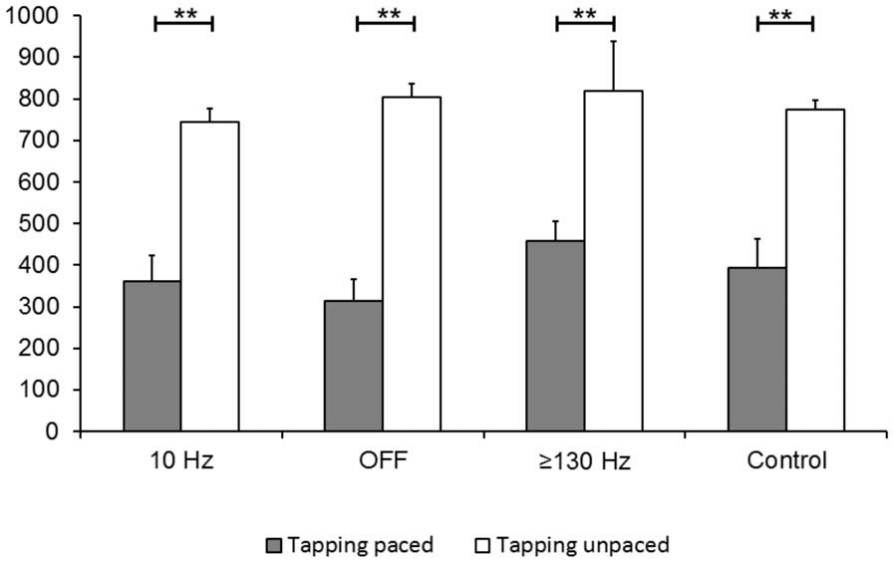
Mean results of tapping. Mean intertap interval in ms (with SEM) of paced and unpaced tapping for controls, PD-patients with stimulation OFF, > = 130 Hz and 10 Hz. Significant difference between paced and unpaced: **p<0.01.

In the tapping task with interstimulus intervals of 800 ms the intertap interval in the unpaced phase was significantly longer (p<0.01) than in the paced phase in all patient conditions and in normal controls. This reflects a strong anticipation in the paced phase (Figure 6). There was no significant difference of mean intervals for the paced phase between controls and patients and within stimulation conditions (mean paced intertap interval in ms: 10 Hz: 360; OFF: 315; > = 130 Hz: 458; controls: 393). Finally, the main dependent variable, the mean intertap intervals in the unpaced phase, did not differ significantly between controls and patients or between different stimulation conditions (interval in ms:10 Hz: 744; OFF: 805; > = 130 Hz: 819; controls: 773). Standard error of mean and standard deviation did also not differ significantly between stimulation conditions and between PD patients and controls in the paced and unpaced phase (paced SD/SEM in ms: 10 Hz: 253/63; OFF: 247/52; > = 130 Hz: 220/48; control: 313/70; unpaced SD/SEM in ms: 10 Hz: 199/44; OFF: 219/49; > = 130 Hz: 367/82; control: 102/23).

Taken together, performance in milliseconds timing, as measured by the time discrimination and tapping tasks, did not differ between patients and controls or between stimulation conditions.

### Reaction time and motor scores

Reaction times were significantly shorter in controls (282±14 ms) than in all patients (10 Hz: 408±35 ms, p<0.01; OFF: 420±20 ms, p<0.001; > = 130 Hz: 321±14 ms, p<0.05). Patients reacted significantly faster in the > = 130Hz stimulation condition than in the OFF state (p<0.05) but there was no significant difference between stimulation frequencies.

Regarding the UPDRS motor score the patient's performances in all conditions was worse than that of the control group (1±0.4; p<0.001). Stimulation conditions differed significantly. In the 10Hz stimulation (45±2, p<0.01) and OFF conditions (49±3, p<0.01) motor performance was worse than in the > = 130 Hz stimulation condition (26±3).

Thus, > = 130 Hz stimulation improved motor performance whereas OFF and 10Hz stimulation did not. As a possible *disease effect* on interval timing correlation between motor score/reaction time in the stimulation OFF-state and 15 sec production and reproduction performance was calculated. However, a significant correlation between motor and interval timing performance could not be detected.

## Discussion

The aim of the study was to examine the impact of 130Hz- and 10Hz STN-DBS on timing functions in PD patients. The main findings were as follows: 1) For interval timing patients and controls over(re-)produced the short intervals of 5 s and under(re-)produced the long intervals of 15 s in both the time production and the reproduction tasks. 2) There was a significantly greater underproduction of 15 s in patients in the stimulation OFF compared to controls, delineating a *disease effect.* 3) There was a significant worsening of time production and reproduction during 10 Hz STN-DBS and a mitigation of time production error during > = 130 Hz STN-DBS for the interval of 15 s. 4) Timing in the milliseconds range was not significantly different between patients and controls or between the different stimulation conditions. Thus, STN-DBS modulates 5 to 15 s interval timing but not millisecond timing in a frequency-dependent manner.

### Methodological consideration

#### Stimulated area

Stimulation contacts yielding optimal motor benefits during chronic stimulation were used. Active contacts were located in the dorso-lateral (motor) part of the STN. It is possible that if a more ventro-medial (associative/limbic) part of the STN had been stimulated impact on timing tasks could have been different. Besides stimulation of the STN per se current spread to the zona incerta or the capsula interna can also be taken into account as a possible mechanism of action.

#### Possible bias: Motor performance, medication influenced motivation, attention, design of the paradigm

Although interval timing tasks required motor action a general effect of motor deficits on these interval timing tasks can be ruled out, as the patients under(re-)produced the 15 s interval - by pressing the reaction button earlier - in the conditions with the worst motor scores (10 Hz and OFF) and longest reaction times. The contrary would be expected if the effects were caused by a motor deficit or by reaction time. All tasks were performed without PD-medication to test solely DBS effects. Long acting dopamine-agonists may have a minimal influence on results. However, 12 hours of L-Dopa withdrawal is the standard regime in clinical testing and was proven to be sufficient to obtain satisfactory results in our previous work [28], [29]. Bias by motivational changes in the non-medicated state can not entirely be ruled out as it was recently shown in D2-receptor overexpressing transgenic mice that modulation of the striatal dopaminergic system can impair timing mediated by cognitive and motivational factors [34]. Another issue might have been fluctuations in the degree of attention during the paradigm. In the production or reproduction paradigm subjects might have been inattentive to the length of the present interval (5s or 15s). This could have lead to enhanced “migration” of performance in both time intervals. Thus, one might argue that e.g. 10 Hz DBS would merely enhance inattentiveness or distractibility rather than affect time processing itself. However, improved cognitive performance in a verbal fluency task during 10 Hz stimulation in our previous experiment [29] argues against this hypothesis. Finally, two issues of the paradigm design can be discussed. First, for the time discrimination task the number of correct judgements was analysed. Another approach was to calculate the difference thresholds (JND) and point of subjective equality (PSE). Such estimates, however, could be noisy due to the given number of 10 presentations per deviation from the standard interval. Nevertheless, we opted not to extend the paradigm as it would have been to demanding for patients in the unmedicated state. Secondly, we did not control for individual counting strategies during interval timing. Therefore the observed effects could be either due to influence of DBS on *timing* or on *counting*. Especially, the relatively good performance during the reproduction task makes counting seem plausible. However, it is assumed that both timing and counting involve brain areas that are influenced by DBS, such as the supplementary motor area (SMA), the inferior frontal gyrus (IFG) or the cingulum, and that counting additionally involves the primary motor cortex, the cerebellum and the putamen [35]. This important fact should be considered in the discussion of time processing effects of DBS.

### Interval Timing: Memory dependent versus memory independent effects

The impact of DBS on interval timing concerning memory dependent tasks such as time *reproduction* and on the “memory migration effect” has been reported before [20]. Our results are in line with these previous findings. Moreover, we provide first evidence for a frequency dependent modulation of time intervals in the range of multiple seconds. The impact of STN-DBS on memory dependent timing functions is presumed to be due to an influence on retrieval of time representations from memory [19]. Thus, it can be concluded that STN-DBS has a frequency dependent modulatory impact on the retrieval of time representations in the range of multiple seconds. In addition, one can assume that this effect increases with higher demand on memory and with the length of the retrieved interval. Therefore this effect is more pronounced in 15 sec rather than in 5 sec. Furthermore we also found time *production* of longer intervals to be modulated in a frequency dependent manner by STN-DBS. This was not expected, as time production was not assumed to be influenced by memory. The time production paradigm was designed to examine the effect of the inner pacemaker on timing functions. It is known that a pathologically slowed internal clock in Parkinsońs disease can be speeded up by L-Dopa [10] and slowed down by dopamine antagonists [36]. However, as performance for long and short time intervals lead to opposite effects our results can't be explained by modulation of an inner pacemaker alone. A confounding influence of memory functions on the production task can't be ruled out, as two different intervals were randomly requested in the task. This hypothesis is supported by recent findings illustrating that impaired interval timing in PD-patients can only be found when intervals with two different durations are tested in one session [37]. This affection of memory in the production task would reflect a stored, possibly semantic memory for intervals needed to provide the target duration. In contrast to this semantic memory for the production task, a working memory mechanism, keeping track of the target stimulus in the reproduction task hast to be considered. Thus, as proposed by the memory migration effect, the long term memory representations for the two time intervals migrated towards each other in the reproduction task rather than in the production task. The fact that patients as well as controls showed a migration of long and short time intervals towards each other indicates that this effect might be a normal working memory phenomenon rather than a pathological phenomenon in PD patients.

### Multi-seconds versus milliseconds timing: Different neural systems depending on the time scale?

In contrast to interval timing in the range of several seconds, milliseconds timing was not significantly modulated by STN-DBS in our study. Therefore, one might conclude that our study supports one classic view, stating that milliseconds timing is not dependent on basal ganglia function and, thus, is not impaired in PD. According to this hypothesis, some authors report that patients with cerebellar lesions have deficits in tapping and time discrimination tasks whereas patients with PD do not have such a deficit [38]. However, this view is not generally accepted and other authors provide evidence suggesting that the basal ganglia are indeed involved in millisecond timing [39], [40]. Especially the striatum seems to be involved in such tasks [41]. Our study design can neither prove nor rule out an involvement of the striatum in milliseconds timing. Nevertheless, we show that milliseconds timing is less vulnerable to electrical stimulation of the STN, presumably as this nucleus forms part of the *indirect* modulatory part of the cortico-striatal-thalamic circuit.

### Impact of subthalamic deep brain stimulation on time representations

In addition to an impact on memory retrieval of time intervals in the range of multiple seconds it is also plausible that DBS affects the comparison and decision processes associated with the retrieval of time representations from memory by affection of the dorsolateral prefrontal cortex (DLPFC). It has been shown that repetitive transcranial magnetic stimulation (rTMS) of the right DLPFC can distort time reproduction of 5 and 15 s intervals [42]. Furthermore imaging studies showed that the right DLPFC is involved in timing functions [43], [44], [45], [46]. Basal-ganglia connections with various cortical areas have been considered in timing functions in a positron emission tomography (PET) study by Jahanshahi et al. [47]. They attribute working memory for time intervals to the left premotor cortex (PMC). Interestingly activation strength of the PMC correlated with the length of the time interval. In our study the DBS effect was mainly seen in the 15 s time interval, which corresponds to the hypothesis that an influence of the PMC is more pronounced by longer intervals. Furthermore, Jahanshahi et al. discuss that the supplementary motor area (SMA) is involved in conscious time representation. An impact of DBS on cortical areas such as the DLPFC, orbital frontal cortex (OFC), SMA and PMC has been shown previously in other tasks besides timing [16], [48], [49]. During cognitive tasks such as verbal fluency DBS deactivates the left inferior-frontal cortex (IFC) [18]. Furthermore, a selective frequency dependent modulation of verbal fluency relying on projections between the STN and frontal cortical areas has been shown in our own previous work [29]. The present study suggests that a frequency dependent modulation of projections between the STN and the DLPFC, PMC and/or SMA might play a key role in the influence on time representations. The frequency modulatory effect on subcortical-cortical networks can be explained by the influence on oscillatory neuronal activity. As 10 Hz stimulation of the STN possibly activates [28] motor parts of a pathological tremor network [27] the current findings might be explained in a similar way. A “coincidence detection” or “striatal beat frequency model (SBF)” [22] postulates that thalamo-cortico-striatal loops are involved in time recognition: striatal neurons detect specific oscillatory activation patterns of frontal cortical areas that are involved in working memory functions. Recordings from single cells support this idea, showing that single cell macaque recordings from the striatum and prefrontal cortex display a temporal interrelation of their firing patterns during time encoding [50]. Striatal recordings in rats during a time reproduction task with a probabilistic reward show selective firing patterns for time intervals of 10 and 40 s [51] and neurons of the prefrontal cortex change their firing rate depending on the number of visually presented items [52]. Thus, the SBF model postulates that frontal cortical representations for the number of items play a role for time recognition. In this sense the SBF model might be used to explain our finding on time processing of longer time durations on a neuronal basis. As the STN is part of the thalamo-cortico-striatal circuit STN-DBS can be interpreted as one example of frequency dependent electrical modulation within the SBF model of interval timing. However, the specific role of 10 Hz with respect to the findings and the model is not known. Nonetheless, we provide first evidence for the possibility of frequency dependent modulation of cognitive time representation in humans by DBS, during which high frequency > = 130 Hz DBS imposes a beneficial timing signal on the basal ganglia and associated areas and 10 Hz DBS further disrupts a system which is already impaired by PD.
